# Global-feature of autoimmune glomerulonephritis using proteomic analysis of laser capture microdissected glomeruli

**DOI:** 10.3389/fimmu.2023.1131164

**Published:** 2023-03-22

**Authors:** Jingjing Dong, Fengping Zheng, Fanna Liu, Jingquan He, Shanshan Li, Wenjun Pu, Huixuan Xu, Zhifeng Luo, Shizhen Liu, Lianghong Yin, Donge Tang, Yong Dai

**Affiliations:** ^1^ Clinical Medical Research Center, The Second Clinical Medical College of Jinan University, Shenzhen People’s Hospital, Shenzhen, Guangdong, China; ^2^ Institute of Nephrology and Blood Purification, The First Affiliated Hospital of Jinan University, Jinan University, Guangzhou, China; ^3^ Department of Nephrology, Peking University Shenzhen Hospital, Shenzhen Peking University-The Hong Kong University of Science and Technology Medical Center, Shenzhen, Guangdong, China; ^4^ Guangxi Key Laboratory of Metabolic Disease Research, The 924th Hospital of the Chinese People’s Liberation Army Joint Logistic Support Force, Guilin, Guangxi, China

**Keywords:** autoimmune glomerulonephritis, proteomic, laser capture microdissection, complement components, IgA nephropathy, lupus nephritis, membranous nephropathy, minimal change nephropathy

## Abstract

**Background:**

IgA nephropathy (IgAN), (LN), membranous nephropathy (MN), and minimal change nephropathy (MCN) are all belonged to autoimmune glomerulonephritis. This study aimed to identify the specific proteomic characteristics of the four GNs diseases in order to provide frameworks for developing the appropriate drug for patients diagnosed with GNs disease.

**Methods:**

Liquid chromatography−tandem mass spectrometry (LC-MS/MS) was utilized to investigate proteomic features of glomerular tissues obtained by laser capture microdissection (LCM). 8 normal control cases, 11 IgAN cases, 19 LN cases, 5 MN cases, and 3 MCN cases in this study were selected for bioinformatics analyses.

**Results:**

The shared overlapping proteins among the top 100 DEPs of each GNs type were mostly downregulated, in which only FLII was significantly downregulated in the four GNs diseases. A2M was significantly upregulated in MN, IgAN, and LN subgroups. The pathway of complement and coagulation cascades was notably activated with NES value ranging 2.77 to 3.39 among MCN, MN, IgAN, and LN diseases, but the pattern of protein expression level were significantly different. In LN patients, the increased activity of complement and coagulation cascades was contributed by the high expression of multiple complements (C1QB, C3, C4A, C4B, C6, C8B, C8G, C9). Meanwhile, both C1QC and C4B were remarkably upregulated in MN patients. On the contrary, complement-regulating proteins (CD59) was substantially decreased in MCN and IgAN subgroup.

**Conclusions:**

The integrative proteomics analysis of the four GNs diseases provide insights into unique characteristics of GNs diseases and further serve as frameworks for precision medicine diagnosis and provide novel targets for drug development.

## 1 Introduction

Autoimmune kidney diseases are caused when the delicate balance between the kidney and immune system is accidentally broken. Targeted specific antigens within the kidney, systemic autoantibody-induced glomerular damage, and circulating immune complex deposition are the three pathophysiological processes of direct/indirect immune-mediated glomerular injuries in glomerulonephritis (GNs) (1). IgA nephropathy (IgAN), lupus nephritis (LN), membranous nephropathy (MN), and minimal change nephropathy (MCN) are the four common autoimmune kidney diseases. More and more studies focus on the potentially shared mechanisms underlying these autoimmune kidney diseases instead of the inter-disease differences, including complement activation initiated by immune complexes, kidney cell damage induced by T-cell infiltration, and inflammatory response (2). However, the development of new drugs for autoimmune kidney diseases in recent years appears slow and extremely difficult. Bioinformatics analyses utilized to explore the potentially shared mechanisms and therapeutic direction of autoimmune kidney diseases are of vital importance to facilitate the development of this field.

The kidney is a complex organ with distinct spatial compartments populated by multiple cell types. To better understand the drivers and the mechanisms underlying complex kidney diseases, we sought to capture different cell types and acquire information on their spatial organization. Unbiased proteomic studies can accurately quantify low abundant proteins and are not constrained by the availability and specificity of antibodies. Meanwhile, the high sensitivity of proteomics technology makes the analysis in tiny tissue samples possible, especially in formalin fixed paraffin-embedded (FFPE) tissue. LCM can be used to isolate specific anatomical microstructure for collecting homogeneous cells (3). Instead of “one-pot” protein extraction and digestion, a combination of LCM and proteomic is able to identify the change of proteomics expression level in specific microstructure and further explain the pathogenesis of human chronic diseases, such as Alzheimer’s disease, cancer, and kidney disease (3–6). Advances in technology combining LCM of glomeruli and mass spectrometry have led to the discovery of novel antigens and a better understanding of the underlying molecular mechanisms of complex kidney disease. In this study, LCM was used to isolate glomerular from FFPE kidney biopsy specimens. Localized proteomics was performed to compare the proteome characteristic relevant to renal pathology of kidney microstructure in IgAN, LN, MN, MCN, and normal control (NC) and further revealed potentially shared molecular mechanisms underlying these four kidney diseases.

## 2 Methods

### 2.1 Patient samples

All participants in this project were collected from Shenzhen People’s Hospital and the First Affiliated Hospital of Jinan University from 2013 to 2020. Based on clinical and histological criteria, 18 patients diagnosed with IgA nephropathy, 21 patients with lupus nephritis, 5 patients with membranous nephropathy, and 5 patients with minimal change disease were included in this study. 11 normal control cases were enrolled. Informed consent was obtained from all participants. This project was approved by the Clinical Research Ethics Committee of the Shenzhen People’s Hospital and the First Affiliated Hospital of Jinan University.

Kidney biopsy specimens were collected for a definitive diagnosis and were then FFPE for the present study. The FFPE sections were cut at 10-μm thickness, deparaffinized in chloroform, and collected onto LCM-compatible slides. LMD 7000 microscope (Leica) with a UV-laser (Power was set for 38, Aperture was 14, Speed was 4) was utilized for microdissection. Renal tissue microstructure, including glomerular, renal interstitial, and renal tubular, were manually selected and dropped into cap tubes due to gravity. Each tube from one case containing 5-8 target microstructures was then stored at −80°C for subsequent LC-MS analysis.

### 2.2 Protein extraction and digestion

The methods of protein extraction and digestion were as follows: (1) Each sample tube containing 5-8 glomerulus of each sample was centrifuged at 20000g for 10 min; (2) The samples were reduced by adding 10 mM Dithiothreitol in 50 mM ammonium bicarbonate at 95°C for 30min; (3) The samples were alkylated with 55mM iodoacetamide and then left to stand in the dark at room temperature for 30 min; (4) The sample was then sonicated for 20 min by using a water bath sonicator; (5) For proteolysis, each sample tube containing the final protein solution was incubated for 16 h at 37°C in 2.5 μg of Trypsin. The digested peptides were next desalted using a Strata X column by 1mL 0.1% FA three times and dried under vacuum.

### 2.3 DDA and DIA analysis by nano-LC-MS/MS

The dried peptide samples were reconstituted with mobile phase A (800ul 75% ACN, 1ml 0.1% FA), and centrifuged at 20,000g for 10 minutes. Then the supernatant was taken for injection. Thermo UltiMate 3000 UHPLC liquid chromatograph was utilized for separation. The sample was enriched, desalted, and then flowed through a tandem self-packed C18 column (150μm internal diameter, 1.8μm column size, 35cm column length). The sample solution was further separated at a flow rate of 500nL/min as a description in the previous study (7). The nanoliter liquid phase separation end was directly connected to the mass spectrometer in the following settings.

For DDA analysis and DIA analysis, LC separated peptides were ionized by nanoESI and injected to tandem mass spectrometer Fusion Lumos (Thermo Fisher Scientific, San Jose, CA) with DDA (data-dependent acquisition) and DIA (data-independent acquisition) detection mode, respectively. The main parameter settings were presented in the previous study (7).

### 2.4 Data processing

DDA data was analyzed using Andromeda search engine within Maxquant, and identification results were used for spectral library construction. Both Oxidation of methionine and protein N‐terminal acetylation was considered as variable modifications. The minimum peptide length was set to seven amino acids. At least one unique peptide was required for the protein identified. The false discovery rate (FDR) threshold for protein and peptide was set to 0.01. The quantitative values of proteins and peptides were obtained from MSstats. The intensity data were log2 transformed and quantile normalized. DEPs were calculated according to the criteria of fold change ≥ 1.5 and P value < 0.05. Comparisons between groups were performed by two-sided Welch’s t-test. Functional annotation enrichments of DEPs were performed by Gene Ontology (GO) analysis and Kyoto Encyclopedia of Genes and Genomes (KEGG) analysis. Gene set variation analysis (GSVA) was conducted by GSVA R package (version 1.30.0). Gene set enrichment analysis (GSEA) is generated from GSEA online tool (http://software.broadinstitute.org/gsea/index.jsp). Protein-protein interaction network and MCODE components were identified using the Metascape online database.

## 3 Results

### 3.1 Baseline clinical features

Low-quality samples with peptides less than 1000 were removed. A total of 46 samples were selected for downstream analyses, including 8 normal control cases, 11 IgAN cases, 19 LN cases, 5 MN cases, and 3 MCN cases in this study.

Clinical information of normal control and autoimmune kidneys group was summarized in **Table 1**. In normal control group, the median age was 37.6 years, and 100% were male. In autoimmune kidneys group, the median age was 33.6 years, and 31.6% were male. The mean 24-hour urine protein level was 2.7 g/24h and the mean hemoglobin level was 108.8g/L in autoimmune kidneys group. The detailed clinical information of normal control and each GNs group was listed in **Table S1**.

**Table 1:** Study population characteristics.

Group	Normal control	Autoimmune kidney diseases
Sample cases	n=8	n=38
Male sex (%)	8 (100%)	12 (31.6%)
Age, yr	37.6 (6.3)	33.6 (13.4)
Hemoglobin (g/L)	NA	108.8 (23.9)
TCHOL (mmol/l)	NA	6.7 (2.4)
BUN (mmol/L)	NA	8.7 (6.4)
Scr (umol/l)	NA	108.8 (62.1)
24-hour urine protein (g/24h)	NA	2.7 (2)
C3 (g/l)	NA	0.8 (0.4)
C4 (g/l)	NA	0.2 (0.1)
Serum IgA (g/l)	NA	2.6 (1.1)
Serum IgG (g/l)	NA	11 (5.8)

### 3.2 Protein identification in each GNs disease

**Figure 1:**
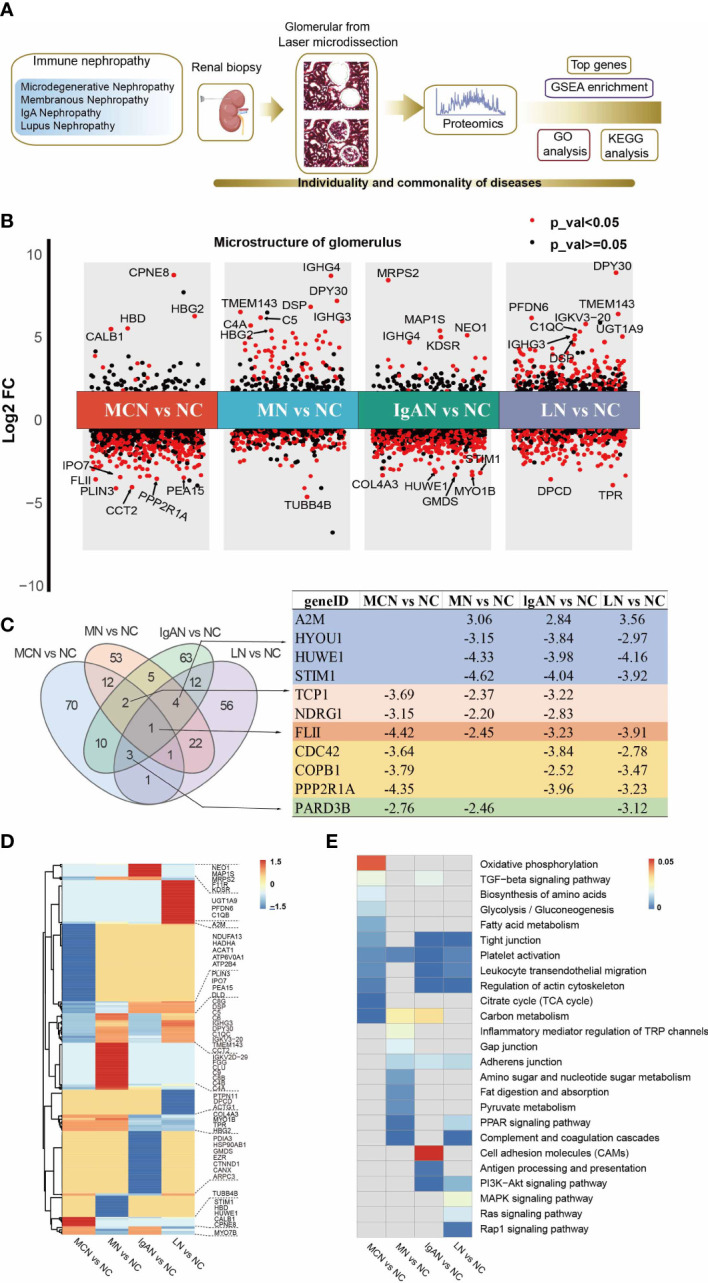
Proteomic analysis of glomerular tissue in four GNs diseases. **(A)** Scheme of the proteomic analysis. **(B)** Differential protein expression analysis showing up- and down-regulated proteins across all four GNs diseases. P value < 0.05 is indicated in red, while P value ≧0.05 is in black. The top 10 of the most significantly DEPs in each cluster were labeled. **(C)** Venn graph representing the overlap of the top 100 ranked identified DEPs in each GNs disease. The characteristic of the shared overlapping proteins that happened in three of four subgroups were presented. **(D)** The heatmap of the top 100 ranked identified DEPs in each GNs disease. **(E)** The heatmap of enriched pathways in the top 100 ranked identified DEPs of each GNs disease using KEGG analysis. The light grey indicates not available (N/A).

The flow chart of the experimental design was shown in **Figure 1A**. For protein expression, we found 1534 proteins in MCN vs NC, 2437 proteins in MN vs NC, 2329 proteins in IgAN vs NC, and 2910 proteins in LN vs NC subgroup were quantitated, shown in **Figure S1A**. We found proteomics data in MCN vs NC was more strongly correlated with that in MN vs NC (R=0.56, P<0.001), while proteomics data in IgAN vs NC was more strongly correlated with that in LN vs NC (R=0.55, P<0.001) rather than other GNs Diseases (**Figure S1B**). The volcano plots of proteomics data of each GNs disease were displayed and the top 10 genes were marked (**Figure 1B**). As shown in the heatmap of the top 100 ranked identified DEPs in each GNs type, there were significant differences within the intra-group of autoimmune kidney diseases (**Figure 1D**). As shown in the Venn diagram (**Figure 1C**), the shared overlapping proteins among the top 100 DEPs of each GNs type were mostly downregulated. Compared with NC group, FLII was significantly downregulated in autoimmune kidneys group. The shared overlapping proteins that happened in three of four subgroups attracted our attention which also may play an important role in the pathogenesis of autoimmune kidney diseases. HYOU1, HUWE1, and STIM1 were significantly downregulated, while A2M was upregulated in subgroups of MN vs NC, IgAN vs NC, and LN vs NC. TCP1 and NDRG1 were downregulated in MCN vs NC, MN vs NC, and IgAN vs NC subgroup. Except for MN vs NC group, CDC42, COPB1, and PPP2R1A were significantly decreased in the other subgroups. And PARD3B was highly reduced groups of MCN vs NC, MN vs NC, and LN vs NC.

We then explored the top 100 DEPs identified in GNs groups which closely related with the clinical parameters including age, weight, Scr, Bun, and 24-hour urine protein (shown in **Table S2**). In MN group, APOA4 was closely linked with the level of Bun and Scr in MN patients. The high expression level of apoA4 have also been observed in diabetic or chronic kidney disease patients with renal function declined faster (8, 9). The expression of MYO1B, GSN, and CDC42 were significantly correlated with Bun and Scr in IgAN and LN patients.

In the group of patients with LN, the pathological indices of activity and chronicity in LN group were calculated and translated the grade into mild, moderate, and severe stage. The protein significantly correlated with activity index or chronic index of LN were listed in **Table S3**.

### 3.3 Functional analysis of top 100 DEPs in each GNs disease

To elucidate the functional roles of the top 100 DEPs in each GNs type, we performed KEGG pathway enrichment analysis (**Figure S2**). Partially selected pathways were presented in **Figure 1E**. The platelet activation pathway was significantly enriched in the four GNs types. In MCN vs NC subgroup, metabolic-related pathways including carbon metabolism, citrate cycle (TCA cycle), fatty acid metabolism, glycolysis/gluconeogenesis, biosynthesis of amino acids, and oxidative phosphorylation, were highly enriched. In MN vs NC subgroup, the top 100 DEPs were significantly enriched in pathways of complement and coagulation cascades, PPAR signaling pathway, and carbon metabolism. In IgAN vs NC subgroup, the enriched KEGG pathways were antigen processing and presentation, leukocyte transendothelial migration, and carbon metabolism. Complement and coagulation cascades, leukocyte transendothelial migration, and MAPK signaling pathway were highly enriched in LN vs NC subgroup.

### 3.4 GSVA analysis in NC group and each GNs diseases

**Figure 2:**
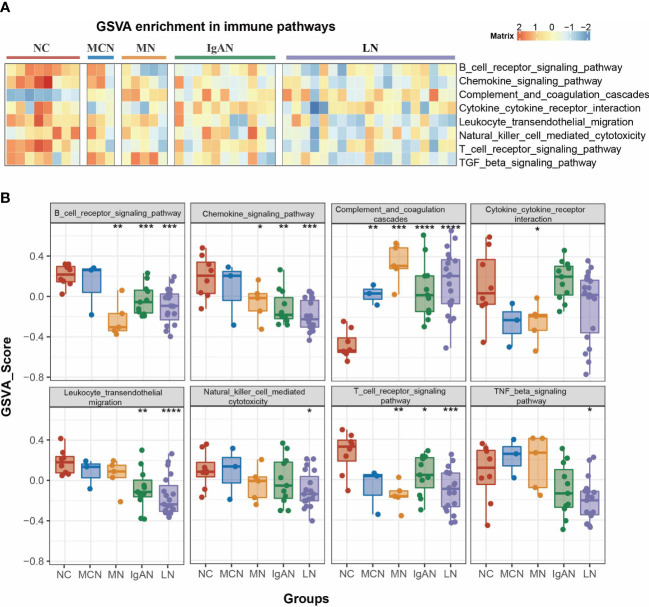
Pathway analysis of four GNs diseases. **(A)** The heatmap of enriched immunity pathways of each clinical sample in the four GNs diseases by GSVA analysis. **(B)** Bar plot representing GSVA score of each clinical sample in the four GNs diseases. Versus NC group; *P < *0.05*, **P < 0.01, ***P < 0.001, ****P < 0.0001.

GSVA of the KEGG pathways was utilized to further calculate pathway activities scored in each clinical case of normal control and GNs disease group. The activity scores of immune-related pathways by GSVA enrichment were visualized in **Figure 2A**. We found a significant change in the activity of 8 immune-related pathways between GNs disease group and normal control group (**Figure 2B**). Complement and coagulation cascades pathway was dramatically activated in the four GNs diseases (all P value <0.01). Chemokine signaling pathway is significantly suppressed in subgroups of MN vs NC, IgAN vs NC, and LN vs NC, while it showed a suppressed trend but without significance in MCN vs NC subgroup. The activity of B cell receptor signaling pathway and T cell receptor signaling pathway is notably decreased in subgroups of MN vs NC, IgAN vs NC, and LN vs NC, instead of MCN vs NC.

### 3.5 GSEA analysis in NC group and each GNs diseases

**Figure 3:**
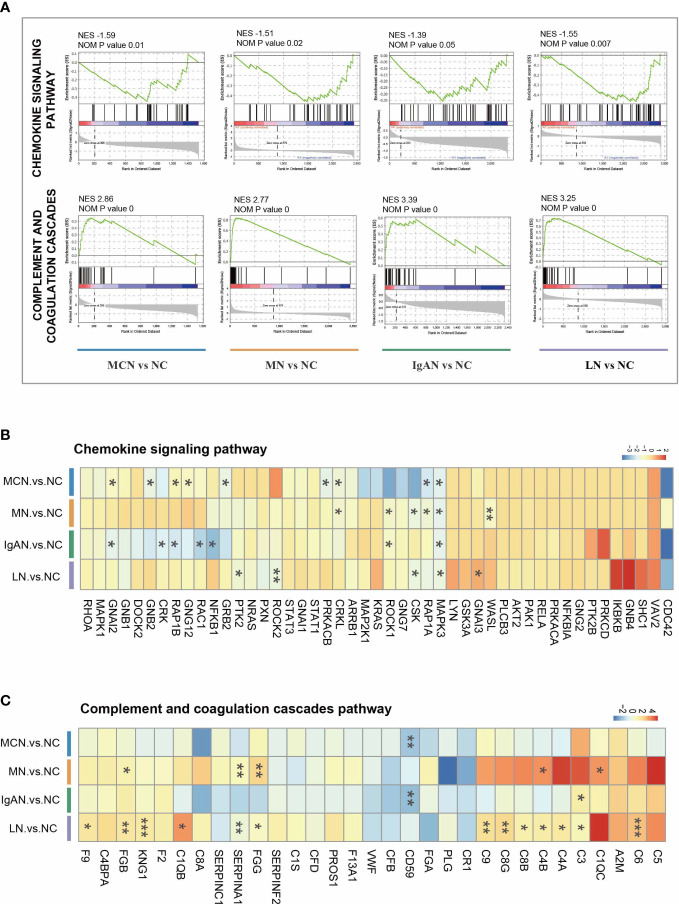
The characteristic of two immunity-related pathways in four GNs diseases. **(A)** GSEA enrichment plots of representative pathways in each cluster. **(B, C)** The heatmap of the fold-change of identified proteins involved in representative pathways. Versus NC group; *P < *0.05*, **P < 0.01, ***P < 0.001.

To exclude the impact of the artificially set thresholds, we also performed GSEA analysis on all identified proteins of proteomics data for functional enrichment. And we focused on the chemokine signaling pathway and complement and coagulation cascades pathway in the four GNs diseases. In comparison to NC group, the chemokine signaling pathway was significantly suppressed with NES value ranging -1.39 to -1.59, while complement and coagulation cascades pathway was dramatically activated with NES value ranging -2.77 to -3.39 in the four autoimmune kidney groups (**Figure 3A**).

Nevertheless, the protein expressions in each GNs disease were markedly distinguished from others (**Figures 3B, C**, **S3**). When comparing MCN to NC group, CRKL, GNAI2, GNB2, GNG12, GRB2, MAPK3, PRKACB, RAP1A, and RAP1B were significantly decreased within chemokine signaling pathway. In MN vs NC subgroup, 6 proteins (CRKL, CSK, MAPK3, RAP1A, ROCK1, and WASL) were decreased. Only 5 proteins (CSK, GNAI3, MAPK3, PTK2, and ROCK2) were highly reduced in the LN vs NC subgroup, while 7 proteins (CRK, GNAI2, MAPK3, NFKB1, RAC1, RAP1B, and ROCK1) were markedly decreased in the IgAN vs NC subgroup. Among all these sharing proteins in the four GNs diseases, only MAPK3 was significantly decreased.

Within complement and coagulation cascades pathway, only a few proteins happened significant changes in MCN and IgAN subgroup with CD59 notably reduced in both two subgroups and C3 upregulated in IgAN subgroup. 5 proteins (C1QC, C4B, FGB, FGG, and SERPINA1) were highly increased in the MN subgroup, while 13 proteins (C1QB, C3, C4A, C4B, C6, C8B, C8G, C9, F9, FGB, FGG, KNG1, and SERPINA1) were markedly increased in the LN subgroup.

### 3.6 Function analysis of sharing DEPs in each GNs diseases

**Figure 4:**
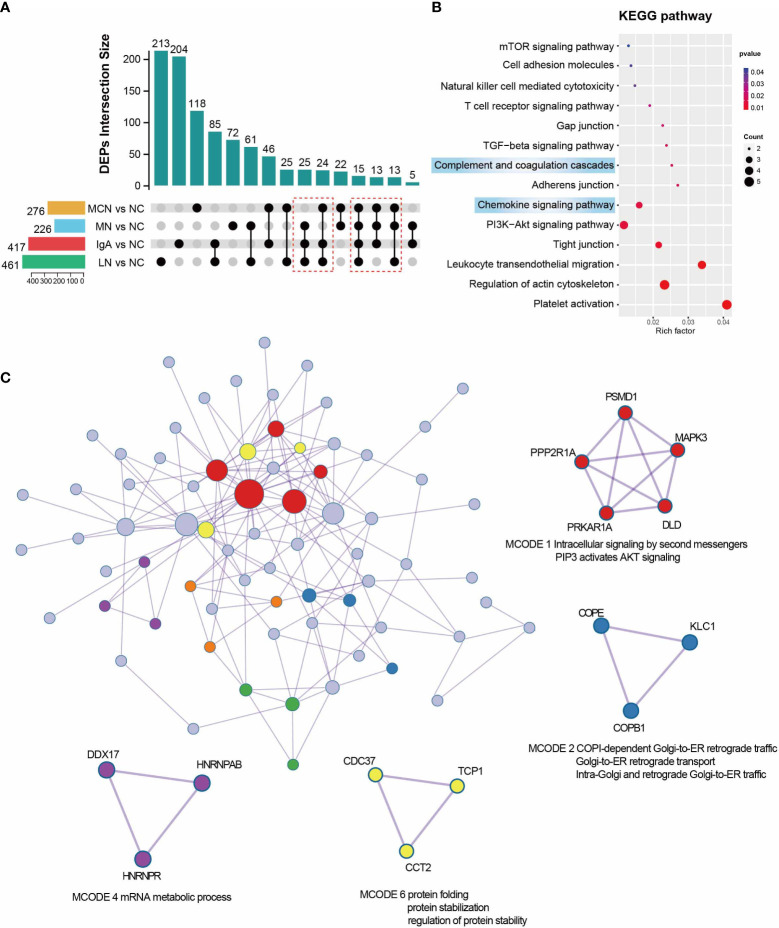
Function analysis of sharing DEPs in Each GNs Diseases. **(A)** UpSet plot showing the overlap proteins of DEGs between NC and four GNs disease groups. in the four GNs Disease. P value < 0.05 and |Log2FC| > 0.585 were used as screening criterion. The sharing DEPs existed in more than three of four GNs disease subgroups were marked with a red box and kept for downstream analysis. **(B)** Enriched pathway of 90 sharing DEPs in GNs diseases using KEGG analysis. **(C)** Protein-protein interaction network and MCODE components were automatically identified in Metascape. MCODE1,2,4, and 6 were marked in different colors and with functional annotation.

For a closer look at the GNs diseases state proteomics, we explored function analysis in sharing DEPs which existed in more than three of four GNs disease subgroups. According to a fold-change threshold of 1.5 and p-value threshold of 0.05, 276 DEPs in MCN vs NC, 226 DEPs in MN vs NC, 417 DEPs in IgAN vs NC, and 461 DEPs in LN vs NC subgroup were identified (**Figure 4A**). 90 sharing DEPs existed in more than three of four GNs disease subgroups were prioritized for further analysis. Platelet activation, leukocyte transendothelial migration, chemokine signaling pathway, and complement and coagulation cascades were highly enriched in KEGG pathway analysis (**Figure 4B**). 6 significant modules were identified from the PPI network constructed by 90 sharing DEPs (**Figure 4C**). PSMD1, MAPK3, PPP2R1A, PRKAR1A, and DLD were involved in MCODE 1 which was defined as Intracellular signaling by second messengers PIP3 activates AKT signaling.

### 3.7 Function analysis of DEPs in NC group and overall GNs diseases

To further explore the common characteristics of the four GNs diseases, we considered these GNs diseases as a whole compared with NC group. Compared with NC group, 115 upregulated proteins (≥1.5-fold) and 152 downregulated proteins (≤0.67-fold) were found in overall GNs diseases group (**Figure S4A**). The top 10 up-regulated proteins in the overall GNs diseases group were APOD, C1QC, IGHG3, IGKV3-20, IGLC3, UGT1A9, KRT16, DSP, C1QB, and KRT6A, while the most significantly down-regulated proteins were P3H1, FERMT2, VKORC1L1, and CUL4A. Among 267 DEPs, the biological process in GO enrichment analysis significantly enriched in immune system process, immune response, and immune effector process (**Figure S4B**). The cellular component was significantly enriched in intracellular, organelle, and cytoplasm (**Figure S4C**). According to molecular function enrichment classification, molecular function, protein binding, and carbohydrate derivative binding were significantly enriched (**Figure S4D**). Partially selected pathways of KEGG analysis were presented in **Figure S4E**. Complement and coagulation cascades, chemokine signaling pathway, platelet activation, and leukocyte transendothelial migration were highly enriched.

## 4 Discussion

To the best of our knowledge, this is the first study to investigate the characteristics of glomerulus proteomics data from normal control group and four GNs diseases, including MCN, MN, IgAN, and LN in parallel. Glomerulus tissues from FFPE were isolated by LCM which promised the purity of our proteomics data and provided a global view of highly complex pathobiology. Herein, we sought to identify the specific proteomic characteristics of each GNs disease. Most importantly, we attempted to elucidate integrative protein expression signatures to explore potential common mechanisms in the immunopathogenesis of MCN, MN, IgAN, and LN disease. Our study would provide a deeper understanding of common GNs-associated pathogenesis and a novel therapeutic direction for autoimmune glomerular disease.

In this study, we found the differential protein expression levels were significantly different in each GNs disease. Functional analysis of top 100 DEPs in each GNs disease was performed. In MCN subgroup, the top 100 DEPs were highly enriched in carbon metabolism, citrate cycle (TCA cycle), fatty acid metabolism pathway. In MN disease, complement and coagulation cascades and PPAR signaling pathway were significantly enriched. The enriched KEGG pathways were antigen processing and presentation and leukocyte transendothelial migration in IgAN subgroup, while complement and coagulation cascades, leukocyte transendothelial migration were notably enriched in LN patients. The parallel comparison among top 100 DEPs in the four GNs diseases showed that FLII was significantly downregulated in the four GNs diseases. As a cytoskeletal protein, FLII is associated with impaired migration, proliferation and adhesion of fibroblasts and keratinocytes (10). It is found FLII expresses in organs susceptible to inflammation and fibrosis, such liver, colon, lung, and kidney (11, 12). Previous studies showed that FLII might impair healing responses and be upregulated in response to tissue inflammation in different inflammatory skin diseases (13). FLII low-expression may result in decreased colon inflammation and notably repress pro-inflammatory cytokine to favor decreased ulcerative colitis severity (12). To date, no study has evaluated the role of FLII in autoimmune glomerular disease. As overlapping DEPs, the data we have presented here indicate that FLII may be a potential therapeutic direction in autoimmune glomerular disease.

In this study the chemokine signaling pathway in the four GNs were significantly suppressed. The protein abundance of MAPK3 (ERK1) in the four GNs were remarkable downregulated. It is known that the mis-localization and signaling imbalances of MAPKs ERK1/2 are closely correlated with cancer to inflammatory disease and may have a significant impact on the outcome of immune kidney diseases (14–16). The specific regulation to ERK1/2 activity appears to significantly ameliorate albuminuria and glomerulosclerosis in immune renal disease (17). The result of parallel comparison in the four GNs showed A2M was significantly upregulated in MN, IgAN, and LN subgroups but without MCN disease. As a known marker of glomerular permselectivity, A2M protein is a nonspecific protease inhibitor and it has been identified as a high-quality urinary biomarker and a strategy for evaluating disease progression of lupus nephritis (18). In multiple kidney diseases, a high concentration of A2M protein in the urine indicates severe glomerular injury. However, very few studies focus on the expression of A2M in kidney tissue. Recent research shows that high A2M mRNA expression of glomerular tissue is associated with a longer time to reach complete remission of proteinuria and poor prognosis of patients with focal segmental glomerulosclerosis (19). In our study, the expression level of A2M in glomerular was increased in MN, IgAN, and LN subgroups but not MCN disease, which may be related to the relatively mild renal pathological changes in MCN disease. Thus, we hypothesized that the different expression of A2M in glomerular among the four GNs may be associated with different clinical prognoses, which could be a potential therapeutic direction for precision therapy in GNs diseases.

The kidney is thought to be particularly vulnerable to damage caused by complement dysfunction (20, 21). The predominant role of kidney in hemofiltration makes it to be the main target of circulating immune complexes, which triggers inflammation and infiltration of immune cells, subsequently, complement activation (22, 23). Accumulating evidence reveals that aberrant complement activation is involved in different kinds of GNs diseases, such as IgA nephropathy (24), lupus nephritis (25), membranous nephropathy (26), and minimal change disease (27). The GSVA result showed the complement and coagulation cascades in the four GNs diseases were aberrantly activated, but the gene expressions involved in this pathway were significantly different. In LN patients, the increased activity of complement and coagulation cascades was contributed by the high expressions of multiple complements (C1QB, C3, C4A, C4B, C6, C8B, C8G, and C9). C1Q, C4A, and C4B regulate the classical complement pathway, while C3 activates alternative complement pathways (28). C3, C4A, C4B, and C5 are powerful chemoattractants that guide some immune cells toward sites of complement activation. C6, C8B, C8G, and C9 are essential genes in the formation of the membrane attacking complex (MAC) C5b-9. In our study, the activated classical and alternative complement pathways, as well as the formation of MAC, subsequently trigger aberrant complement activation and participate in the initiation and progression of SLE (23). The gene expression model in MN subgroup was similar to SLE patients. An intense stimulation of multiple complements (C1QC, C4B, C3, C4A, C5, C6, C8B, C8G, C9) was observed in MN patients wherein both C1QC and C4B are remarkably up-regulated. It is known that C5 is not a stable molecule cleaved by C5 convertase into the C5a, and C5b. C5a further leads to the activation of eosinophils and neutrophils and triggers pro-inflammatory effects, and C5b participates in the formation of the MAC C5b-9 to induce cell injury and kidney diseases (29, 30). High level of C5b-9 complexes was found in patients diagnosed with immune Glomerulus diseases (31). In podocytes, sublytic C5b-9 may activates downstream pathways including protein kinases, reactive oxygen species, growth factors/gene transcription, endoplasmic reticulum stress, and the ubiquitin-proteasome system, and further disrupt the integrity of the cytoskeleton and slit diaphragm, causing the appearance of massive proteinuria (31, 32). In nephritis rat models, sublytic C5b-9 induces glomerular mesangial cell proliferation *via* activation of ERK1/2, SOX9, and Cyclin D1 (16). The expression of C5 was indeed upregulated in LN, IgAN, and MN groups, despite cannot distinguish the abundance of C5a and C5b from C5 is our study. Specifically targeting C5 to regulate the complement process may be a potential and interesting direction in GNs diseases. However, distinct gene expression patterns were found in MCN and IgAN subgroups. Instead of increasing multiple complements, the protective factor (CD59) was substantially attenuated in those groups. CD59, as a membrane-bound complement regulator, binds C8 and C9, prevents the recruitment of C9 to the C5b-8 complex, and thereby inhibits the formation of MAC (33). Our results supported the attenuated protective factor (CD59) in the complement and coagulation cascades pathway was closely associated with the pathology of MCN and IgAN disease. The selective target complement components are considered as a potentially valuable and promising direction (27, 34). Advances in understanding the common characteristics inter-GNs diseases and specific proteomics features intra-GNs diseases might offer more targeted and accurate therapeutic interventions for these autoimmune kidney diseases. Indeed, our overall findings strongly support that the complement and coagulation cascades was aberrantly activated in diverse kidney diseases, including MCN, MN, IgAN, and LN, but the individually pathogenetic proteins change were likely to be unique to each disease. SLE and MN subgroups share a similar gene expression pattern that multiple pathogenetic complements were significantly increased. In contrast, a different gene expression type with protective factor (CD59) attenuating was found in IgAN and MCN patients.

There are some limitations in our research. First, the small number size of clinical samples, especially in MCN and MN subgroup, limit the representativeness and generalizability of our findings. Second, the patients included in this research were Asian from one single center. Three, all the normal controls in our study are male, and the effect of sex on the proteomics data cannot be assessed. However, this work is a cross-sectional study with the similar and different characteristics among the four GNs diseases, which indeed provides new insights into our understanding of GNs.

## 5 Conclusion

In summary, our study generated a comprehensive proteomics analysis of the four GNs, including MCN, MN, IgAN, and LN, based on glomerular tissues obtained by LCM technology. The parallel comparison among top 100 DEPs in the four GNs diseases showed that FLII was significantly downregulated in GNs diseases. The aberrantly activated complement and coagulation cascades were found among MCN, MN, IgAN, and LN disease. A multitude of damage-associated complement and coagulation factors were upregulated in SLE and MN subgroups, while complement-regulating proteins (CD59) were decreased in IgAN and MCN patients. As the complement components linked to GNs are targeted by emerging therapeutics, this integrative analysis provides insights into unique characteristics of GNs diseases and further serve as frameworks for precision medicine diagnosis and provide novel targets for drug development.

## Data availability statement

The data presented in the study are deposited in the CNGBdb repository, accession number CNP0004014.

## Ethics statement

The studies involving human participants were reviewed and approved by LL-KY-2020167 KY-2020-034. Written informed consent to participate in this study was provided by the participants’ legal guardian/next of kin.

## Author contributions

JD and FZ contributed to the writing of the original draft and preparation. LY, DT, and YD contributed to the conceptualization. JH contributed to the methodology. SSL, SZL, and ZL participated in the collection of tissue samples and clinical data. FL contributed to the project administration. WP and HX contributed to the preparation of the manuscript. All authors contributed to the article and approved the submitted version.
