# Supplementary material for: Global-feature of autoimmune glomerulonephritis using proteomic analysis of laser capture microdissected glomeruli
Source: Front Immunol. 2023 Mar 22;14:1131164. doi: 10.3389/fimmu.2023.1131164 (PMC10077062; doi:10.3389/fimmu.2023.1131164)

# Supplementary Material

**Global-Feature of Autoimmune Glomerulonephritis Using Proteomic Analysis of Laser Capture Microdissected Glomeruli**

Jingjing Dong ^1,2#^, Fengping Zheng^1,3#^, Fanna Liu^2^, Jingquan He^1^, Shanshan Li^1^, Wenjun Pu^1^, Huixuan Xu^1^, Zhifeng Luo^4^, Shizhen Liu^2^, Lianghong Yin^2^*, Donge Tang^1^*, Yong Dai^1,4^*

^1^ Clinical Medical Research Center, The Second Clinical Medical College of Jinan University, Shenzhen People’s Hospital, Shenzhen, Guangdong 518020, China

^2^ Institute of Nephrology and Blood Purification, the First Affiliated Hospital of Jinan University, Jinan University, Guangzhou 510632, China

^3^ Department of Nephrology, Peking University Shenzhen Hospital, Shenzhen Peking University-The Hong Kong University of Science and Technology Medical Center, Guangdong 518036, China

^4^ Guangxi Key Laboratory of Metabolic Disease Research, the 924th Hospital of the Chinese People's Liberation Army Joint Logistic Support Force, Guangxi 541002, China

Table S1 Patient demographics, clinical data, and biospy class.

| **Supplementary Table 1. Patient demographics, clinical data, and biospy class.** | | | | | | | | | | | | |
| --- | --- | --- | --- | --- | --- | --- | --- | --- | --- | --- | --- | --- |
| **Group** | Patient_ID | Gender | Age (year) | Hemoglobin (g/L) | TCHOL (mmol/l) | BUN (mmol/L) | Scr (umol/l) | 24-hour urine protein(g/24h) | C3 (g/l) | C4 (g/l) | Serum IgA (g/l) | Serum IgG (g/l) |
| **Normal Control** | N1 | Male | 39 | NA | NA | NA | NA | NA | NA | NA | NA | NA |
|  | N2 | Male | 32 | NA | NA | NA | NA | NA | NA | NA | NA | NA |
|  | N3 | Male | 34 | NA | NA | NA | NA | NA | NA | NA | NA | NA |
|  | N4 | Male | 37 | NA | NA | NA | NA | NA | NA | NA | NA | NA |
|  | N5 | Male | 47 | NA | NA | NA | NA | NA | NA | NA | NA | NA |
|  | N12 | Male | 39 | NA | NA | NA | NA | NA | NA | NA | NA | NA |
|  | N16 | Male | 46 | NA | NA | NA | NA | NA | NA | NA | NA | NA |
|  | N17 | Male | 27 | NA | NA | NA | NA | NA | NA | NA | NA | NA |
| **Minimal Change Nephropathy** | P2 | Female | 21 | 128 | 9.41 | 2.3 | 66 | 4.56 | 1.5 | 0.41 | 3.55 | 4.81 |
|  | P5 | Male | 21 | 152 | 14.61 | 4.3 | 63 | 5.34 | NA | NA | NA | NA |
|  | P6 | Male | 16 | NA | NA | NA | NA | NA | NA | NA | NA | NA |
| **Membranous Nephropathy** | M2 | Male | 52 | 97 | 7.09 | 6.2 | 120 | 4.76 | 1.47 | 0.28 | 1.3 | 3.26 |
|  | M3 | Male | 57 | 92 | 7.47 | 19.4 | 259 | 7.82 | 0.95 | 0.33 | 2.94 | 11.19 |
|  | M4 | Female | 48 | 130 | 8.2 | 2.7 | 60 | 1.72 | 0.84 | 0.31 | 1.74 | 3.87 |
|  | M5 | Male | 57 | 158 | 9.77 | 6.1 | 120 | 1.6 | 1.12 | 0.35 | 1.13 | 3.56 |
|  | M1 | Female | 46 | 139 | 8.22 | 4.1 | 66 | 2.71 | 1.26 | 0.36 | 2.01 | 8.76 |
| **IgA Nephropathy** | I4 | Female | 41 | 132 | 8.68 | 27.06 | 289 | 1.498 | 1.116 | 0.32 | 1.78 | 8.71 |
|  | I6 | Female | 32 | 129.8 | 5.58 | 2.96 | 51.6 | 0.613 | 1.141 | 0.241 | 3.22 | 11.03 |
|  | I7 | Male | 24 | 126 | 5.52 | 6.5 | 75 | 1.87 | 0.83 | 0.21 | 5.14 | 12.9 |
|  | I9 | Female | 36 | 114 | 7.2 | 2.59 | 49 | 1.86 | 1.366 | 0.406 | 2.32 | 8.2 |
|  | I10 | Female | 27 | 119.6 | 4.15 | 1.41 | 48.7 | 1.085 |  |  |  |  |
|  | I12 | Male | 19 | 145.5 | 4.43 | 4.47 | 67.6 | 0.032 | 1.53 | 0.445 | 3.86 | 13.51 |
|  | I13 | Female | 34 | 132 | 4.74 | 3.9 | 73 | 0.31 | 1.3 | 0.22 | 2.76 | 9.73 |
|  | I15 | Female | 29 | 114 | 4.17 | 3.7 | 51 | 0.346 | 1.154 | 0.319 | 3.58 | 13.82 |
|  | I3 | Male | 50 | 119 | 4.02 | 10.49 | 129 |  | 1.112 | 0.443 | 4.48 | 14.07 |
|  | I4_20 | Female | 45 | 100 | 4.37 | 3.18 | 58 | 0.035 | 0.896 | 0.301 | 3.9 | 7.23 |
|  | I8 | Female | 50 | 100 | 2.53 | 10.28 | 119 | 0.009 | 0.952 | 0.254 | 3.48 | 17.77 |
| **Lupus Nephritis** | L11 | Female | 28 | 80 | 7.91 | 19.9 | 162 | 3 | 0.61 | 0.15 | 1.36 | 2.54 |
|  | L12 | Male | 44 | 115 |  | 3.3 | 94 | 2.5 | 1.23 | 0.15 | 3.86 | 12.87 |
|  | L13 | Female | 23 | 84 | 4.91 | 15.7 | 221 | 2.38 | 1 | 0.2 | 2.65 | 13.65 |
|  | L14 | Female | 16 | 102 | 3.1 | 5.5 | 47 | 0.71 | 0.04 |  | 2.43 | 20.24 |
|  | L15 | Female | 26 | 87 | 5.42 | 10.6 | 191 | 5.66 | 1.09 | 0.32 | 3.13 | 16.53 |
|  | L16 | Female | 39 | 80 | 6.45 | 10.1 | 196 | 2.91 | 0.16 | 0.02 | 4.34 | 22.08 |
|  | L18 | Female | 24 | 75 | 5.37 | 15.4 | 148 | 1.54 | 0.14 | 0.01 | 2.13 | 10.66 |
|  | L19 | Male | 18 | 93 |  | 14 | 105 | 2.57 | 0.2 | 0.01 | 1.47 | 26.3 |
|  | L20 | Female | 12 | 120 | 7.85 | 6.2 | 49 | 2.89 | 0.73 | 0.15 | 1 | 2.29 |
|  | L22 | Male | 25 | 128 | 5.29 | 6 | 89 | 4.53 | 0.6 | 0.02 | 1.8 | 19.99 |
|  | L23 | Female | 43 | 95 | 5.66 | 5.7 | 95 | 4.02 | 0.36 | 0.06 | 2.7 | 11.85 |
|  | L24 | Female | 17 | 72 | 7.5 | 13 | 210 | 5.51 | 1.04 | 0.19 | 2.07 | 5.12 |
|  | L25 | Female | 18 | 54 | 8.97 | 8.2 | 112 | 1.28 | 0.18 | 0.08 | 1.97 | 5.08 |
|  | L26 | Male | 49 | 86 | 9.24 | 25.9 | 151 | 4.41 | 0.307 | 0.033 | 3.07 | 14.74 |
|  | L1 | Female | 54 | 122 | 6.5 | 4.16 | 50 | 4.3 | 0.42 | 0.04 | 2.41 | 10.16 |
|  | L2 | Female | 49 | 84 | 9.78 | 9.32 | 73 | 0.7 | 0.54 | 0.25 | 3.75 | 11.23 |
|  | L3 | Female | 41 | 112 | 8.89 | 13.23 | 102 | 3.15 | 0.59 | 0.34 | 2.56 | 4.89 |
|  | L4 | Female | 28 | 90.4 | 5.23 | 8.43 | 101 | 6.15 | 0.52 | 0.17 | 1.51 | 8.18 |
|  | L5 | Female | 19 | 119.2 | 6.05 | 5.1 | 65.9 | 2.02 | 0.31 |  | 1.05 | 15.77 |

Figure S1 The characteristic of proteins identified in IgAN, LN, MN, and MCN subgroups.


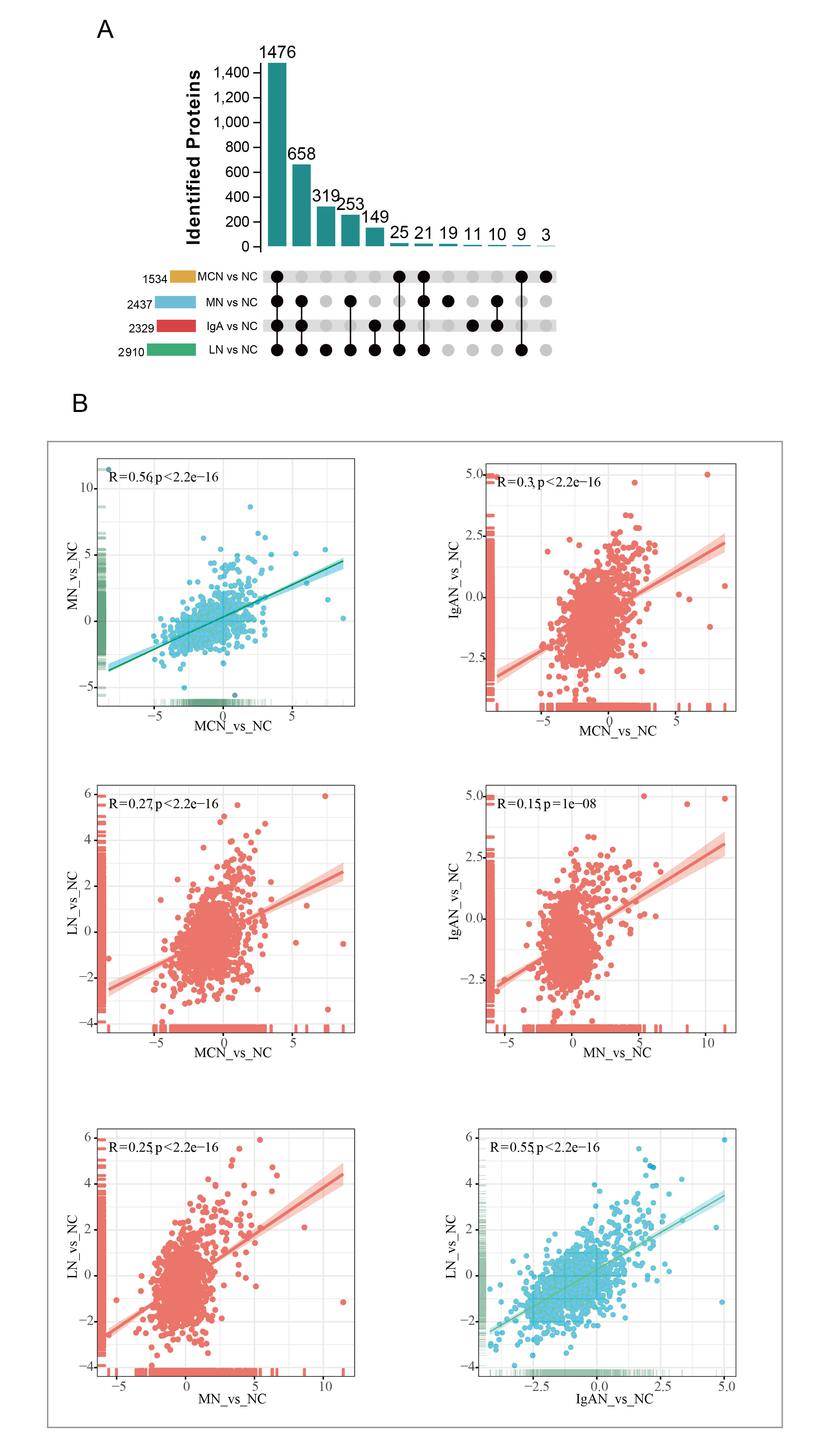


Figure S2 KEGG pathway enrichment analysis of top 100 DEPs in IgAN, LN, MN, and MCN subgroups.


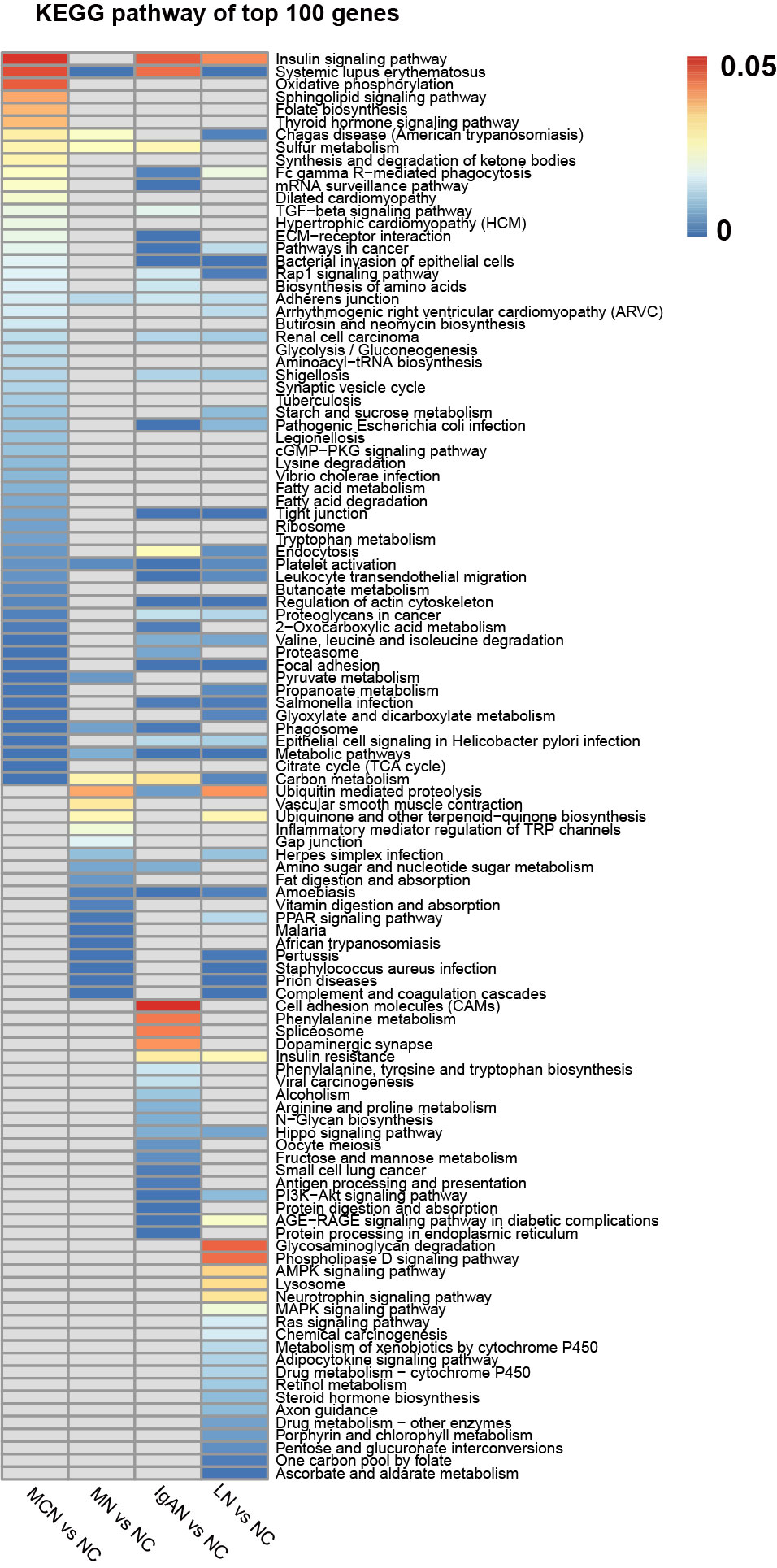


Figure S3 The protein expressions of Chemokine signaling pathway and Complement and coagulation cascades pathway in each GNs disease.


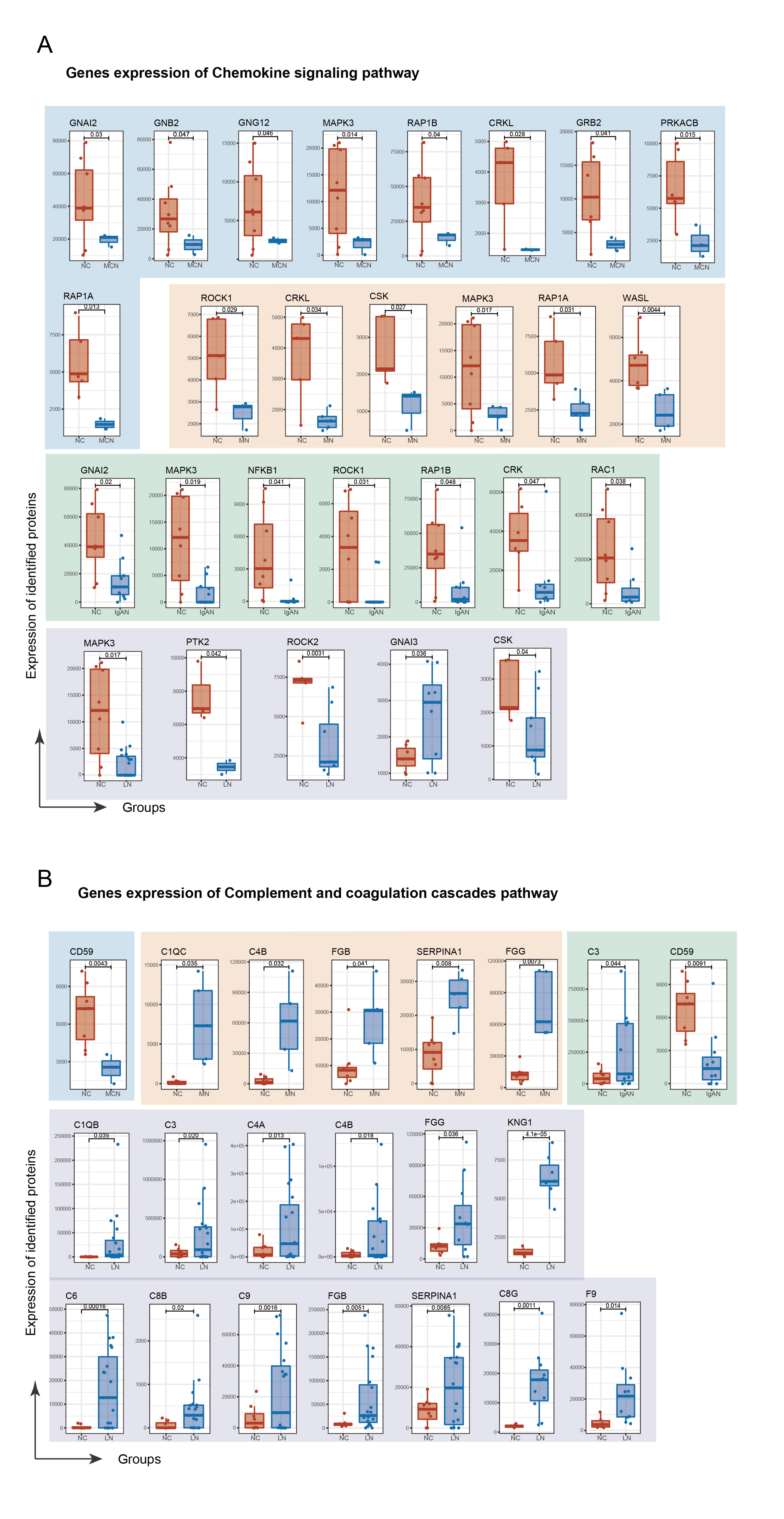


Figure S4 Function analysis of DEPs in NC group and overall GNs diseases.


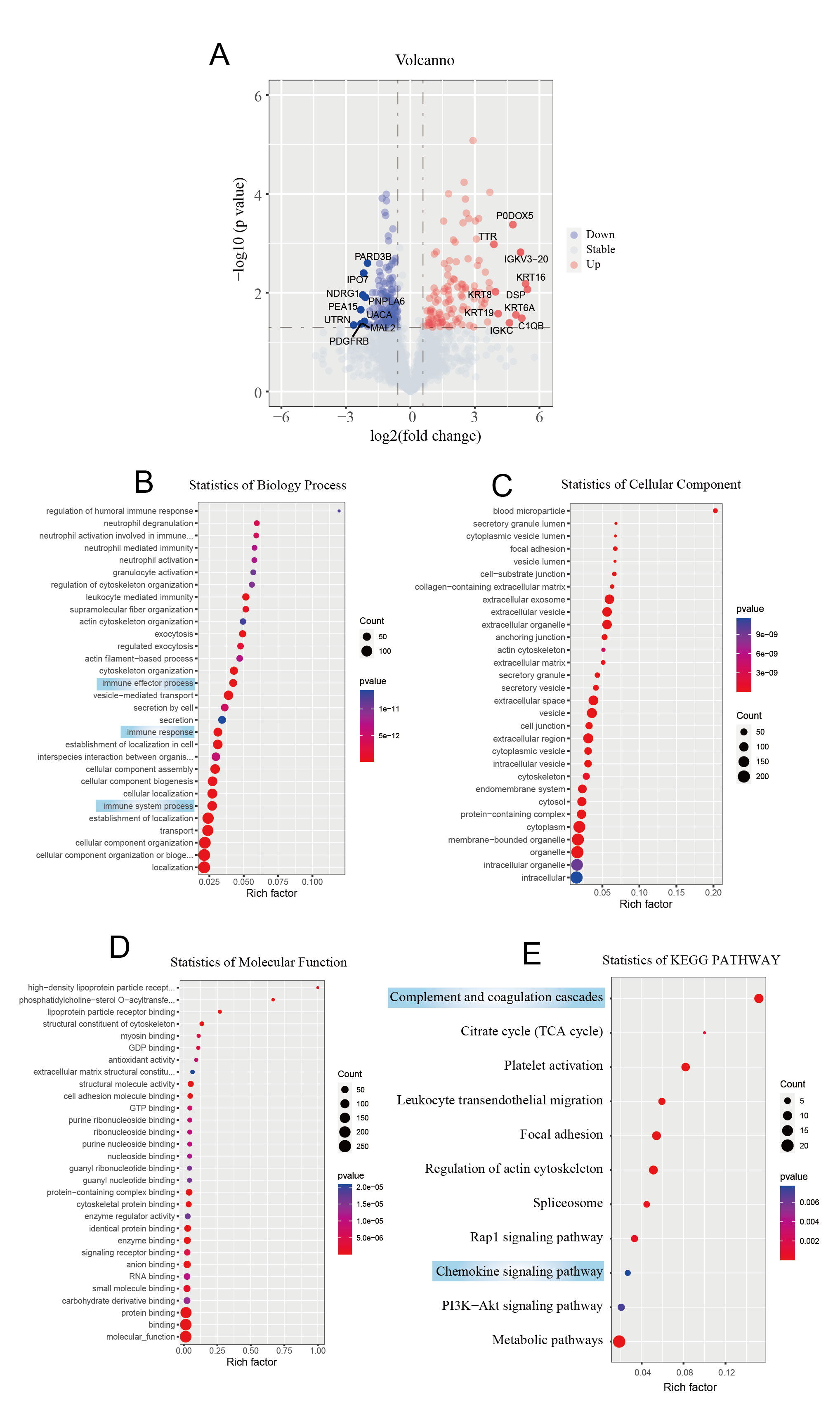

Supplement: Supplementary file 1 [file DataSheet_1.doc]
